# Mixed Incontinence: How Best to Manage It?

**DOI:** 10.1007/s11884-012-0161-8

**Published:** 2013-01-11

**Authors:** Massimo Porena, Elisabetta Costantini, Massimo Lazzeri

**Affiliations:** Department of Medical-Surgical Specialties and Public Health, Urology and Andrology Section, University of Perugia, Ospedale S. Maria della Misericordia. Loc. S. Andrea delle Fratte, Perugia, 06100 Italy

**Keywords:** Urinary incontinence, Mixed urinary incontinence, Epidemiology, Prevalence, Quality of life, Pathophysiology, Urethrovesical axis, Intrinsic sphincter deficiency, Integral theory, Diagnosis, Urodynamic assessment, Therapy, Pelvic floor muscle exercises, Biofeedback, Duloxetin, Oestrogens, Antimuscarinics, Surgery, Pubovaginal sling, Colpo-suspension, TOT, TVT

## Abstract

Although common in women, mixed urinary incontinence (MUI) is under-reported and under-treated. It is linked to concomitant disturbances, which may be due to childbirth, ageing, or other medical conditions, in the complex bladder-urethra coordinated system of urine storage and emptying. Primary care physicians can evaluate MUI through history and simple clinical assessment or they can avail of more complex device and tools, such as urodynamic assessment. There is a wide range of therapeutic options. The recent proliferation of new drug treatments and surgical devices for urinary incontinence offers innovative strategies for therapy but products risk being introduced without long-term safety and efficacy assessment. Direct-to-consumer advertising has increased public awareness of MUI.

## Introduction

Mixed incontinence, usually considered as a combination of stress incontinence and urgency incontinence, is highly prevalent in everyday clinical practice. Several physicians fail to take account of wide variation in the relative importance of the stress and urgency components experienced by patients. The lack of clarity awareness regarding the real clinical picture makes diagnosis and management extremely difficult. The purpose of this paper is to improve bedside awareness regarding women with mixed urinary incontinence providing a snapshot over definitions, diagnosis and treatment by pointing out the traditional and recent advances in treatment options.

## Definition

The International Continence Society’s (ICS) standardized nomenclature of MUI is: complaint of involuntary leakage associated with urgency and also with exertion, effort, sneezing or coughing [1]. When urodynamic assessment is performed, MUI is represented by stress incontinence and detrusor overactivity (DO) with or without incontinence. Unfortunately, in daily clinical practice symptomatic and urodynamic definitions fail to take wide variations into account. Generally speaking patients fall into two main categories: those with urodynamic stress incontinence and DO with incontinence (OAB wet) and those with urodynamic stress incontinence and DO without incontinence (OAB dry). We should also bear in mind the following definitions: clinical MUI, based only on clinical evaluation and urodynamic MUI. Finally, a proposal was made to use the terms “mixed incontinence”, when incontinence was objectively shown and “mixed symptoms of incontinence” when it was subjectively reported by patients.

The definitions of MUI encompass different aspects of the same kind of incontinence. The Urinary Incontinence Treatment Network upheld the view that MUI definitions did not adequately categorize clinically relevant UI subgroups [2•]. Indeed, no definition provides answers to the questions as to what the predominant type is or what the most bothersome type is in the patient’s perspective. Semantic definitions of urge predominant MUI or stress predominant MUI were introduced to facilitate practical use and orient treatment.

## Epidemiology

A large, population-based survey of USA women aged 30–90 years, revealed about 45% prevalence of urinary incontinence of any kind/any type and half the incontinent women had MUI symptoms [3]. The NOBLE survey estimated that 5.2 million adults aged >18 years had mixed incontinence [4]. In a Norwegian questionnaire-based estimate, the prevalence of urinary incontinence was 25%, with 36% MUI [5]. Confirming American findings [3] Minassian et al., observed the overall prevalence of MUI in women was 14.5%, with 57% reporting severe MUI incontinence compared with 36% and 37% of women with stress (SUI)- or urge (UUI)-only urinary incontinence [6]. The prevalence rates of urinary incontinence subtype varied with responders’ age, ethnicity, how the question was asked, and how the subtype was defined [4, 7, 8]. Patients with MUI and UUI scored worse than those with SUI but no significant differences were found in HRQoL scores in patients with MUI and UUI, meaning that urgency in mixed incontinence has a greater impact on quality of life than stress [9].

## Pathophysiology

Normal continence in women is a complex coordination of bladder, urethra and pelvic muscles as well as surrounding connective tissues. MUI is caused by disturbances in storage and emptying. Urethral sphincter dysfunction and bladder dysfunction may coexist in individuals, and insisting that patients fit into one specific category could compromise clinical care. Four pathophysiological theories to account for SUI dominate the literature:

i) **Alterations in the urethrovesical axis**: incontinence depends on a sudden, abnormal displacement of the urethra and the urethro-vesical junction immediately behind the pubic symphysis. This theory is supported by evidence that anatomy, topography and mutual spatial relationships are essential for district function. However, since the urethral axis at rest, during bearing down and in its total excursion, was not found to be significantly different in continent and incontinent women, the urethral axis and its sphincter function could not be correlated [10].
ii) **Intrinsic sphincter deficiency**: In 1977 McGuire suggested that SUI depended on a sphincter mechanism deficiency. Loss of intrinsic sphincter integrity together with age-dependent rabdosphincter apoptosis could play major roles in the development of female incontinence. Unfortunately, evidence does not fully support this theory as 51% of continent climateric women had bladder neck incompetence and 21% of continent nulliparous women had an open bladder [11].
iii) **Hammock theory:** In 1994 DeLancey reported that the urethra lay on a supportive layer of endopelvic fascia and anterior vaginal wall and was stabilized by its lateral attachments to the arcus tendineus fascia and levator ani. Urethral closure depends on urethral compression against the rigid support of pubocervical fascia and anterior vaginal wall. MRI findings supported the theory as they confirmed thinning of the urethra, a high degree of puborectalis asymmetry and distortion of periurethral, pubourethral and paraurethral ligaments in incontinent women [12, 13]. Post-mortem and other anatomical studies, however, found three-fold variations in levator ani muscle thickness, endopelvic fascia and urethra thickness were also present in continent women [14].
iv) **Integral theory:** More recently Petros and Ulmsten argued that “*the laxity of the anterior vagina wall leads to activation of stretch receptors in the bladder neck and proximal urethra, resulting in dissipation of urethral closure pressure and inappropriate activation of micturition reflex*”. This last theory has been advocated to explain both SUI and MUI.

The pathophysiology of MUI needs to involve factors such as striated muscle atrophy, oestrogen status, histomorphological abnormalities and ultrastructural changes, all of which are under study. Although beyond the scope of the present article, it is important to mention that women with MUI may have decreased vaginal collagen content, increased vaginal mRNA matrix metalloproteinases and a greater collagen breakdown [15].

The pathophysiology of urgency/OAB and UUI remains poorly understood. UUI and OAB may be due to afferent dysfunction, CNS incapacity to handle information and evoke adequate answers as well as independent activity of the periphery.

In conclusion, one theory or one risk factor is insufficient to explain the complexity of MUI. Consequently, the “trampoline theory” attempts to encompass all the factors that may play a role in MUI [16]. Although malfunction of one element in the continence mechanism rarely results in MUI, malfunction of several elements and inability to compensate for loss of function, means the” trampoline” will malfunction.

## Therapy

MUI treatment consists of conservative management, pharmacotherapy and surgery.

### Conservative Management

Conservative management comprises any therapy that does not involve pharmacological or surgical intervention. It can be initiated after simple assessment in many patients and should be considered the mainstay of primary care treatment of women with MUI. It includes lifestyle interventions, bladder retraining, anti-incontinence devices, biofeedback, complementary therapies and pelvic floor muscle exercises (PFME). The goals of behavioral training are to correct voiding patterns, improve the ability to suppress urge and increase bladder capacity and continence. The benefits of weight loss are supported by level 2 evidence in morbidly obese patients and level 1 evidence in moderately obese patients [17]. No data have been reported on the impact of smoking cessation on incontinence. Evidence about caffeine, alcohol or fluid intake is conflicting. As obesity is known to be a modifiable risk factor for SUI, Subak et al. investigated whether weight loss was effective treatment of MUI [18•]. In a randomized controlled trial, 338 overweight and obese women, most of whom are suffering from MUI with urinary incontinence, were randomized to an intensive weight-loss program and behavior modification or to a structured education program. After 6 months women in the weight-loss program lost significantly more weight and had significantly fewer incontinence episodes weekly than those in the education group.

Grade A evidence recommendation supports use of PFME in women with MUI [17] as the overall cure/improvement rates ranged from 56 to 70%.

### Pharmacotherapy

Since MUI has been regarded as two coexisting disorders (SUI/UI), first-line drugs help both conditions, with treatment focusing on the pathophysiology of predominant symptoms. The main drug strategies consist of hormone replacement, drugs targeting SUI, drug targeting UUI and drugs with a balanced activity over SUI and UUI.

In the treatment of SUI and MUI data are contradictory on the efficacy of topical and systemic oestrogens. Even though the Cochrane review (15 RCT studies) showed that incontinence improved with hormone replacement therapy (HRT) [19], oral or transdermal oestrogen may increase the risk of incontinence [20]. The International Consultation on Incontinence gave oestrogen a grade D recommendation and advised against using it as treatment for incontinence.

Some studies of antimuscarinic drugs and SNRIs (serotonin/norepinephrine reuptake inhibitors) suggested that each substance had a positive effect on MUI [21]. Although no robust data support the effectiveness of Imipramine in MUI, it was recommended because of its dual action of anticholinergic and noradrenergic or serotonergic reuptake inhibition. It has low morbidity and variable success but may be useful in young and elderly women who are not candidates for surgery. Imipramine received a grade D recommendation for the treatment of SUI.

Duloxetine was the first drug the EMA specifically approved for the treatment of SUI. In a preliminary study in women with MUI, median MUI episodes were reduced by 62% at 40 mg/daily and by 63% at 80 mg/daily. In a randomized, placebo-controlled, double-blind clinical trial Bent and colleagues randomized 588 women with MUI to 80 mg daily duloxetine or placebo [22]. Overall, episodes of frequency, stress and urge incontinence were significantly fewer with duloxetine. Similar findings were reported in elderly women with stress predominant MUI [23].

Evidence supports the use of antimuscarinic agents (oxybutynin, tolterodine, solifenacin and fesoterodine) in MUI as well as in OAB [24]. Since most studies considered subgroups of patients with MUI within studies on OAB, few investigations specifically targeted their effects on MUI. Karram and Bhatia reported that 32% of 52 patients with SUI and UUI were cured and 28% improved [25]. Dmochowsky et al. found that 3.9 mg transdermal oxybutynin significantly improved symptoms in patients with MUI [26]. Goepel found that symptoms regressed in 60% of 410 patients with MUI [27]. Kreder et al. compared the efficacy of tolterodine twice daily in 239 patients with urge-predominant MUI and 755 patients with UUI alone, reporting no significant between-group differences, with dry rates of 39% and 44%, respectively [28]. In the Mixed Incontinence Effectiveness Research: Investigating Tolterodine (MERIT) study, 854 women with urge predominant MUI were randomized to 4 mg extended release (ER) tolterodine or placebo for 8 weeks [29]. There were 12.3 weekly incontinence episodes fewer in patients undergoing active treatment and eight episodes fewer in those taking placebo. In a prospective, randomized study with solifenacine (5–10 mg), 1041/2696 women (39%) with urgency-predominant MUI achieved median reductions in incontinence episodes of 82% (5 mg) and 94% (10 mg) vs 64% placebo. Urgency episodes were reduced by a median of 73% (5 mg) and 69% (10 mg) vs. 42% placebo [30].

### Surgery

Since surgery is used mainly to treat SUI, primary physicians, urologists and gynecologists should include the option of surgery during treatment counseling for women with predominant SUI. Once surgery is decided, a consultation with a dedicated surgeon is mandatory so as to offer the most choices to the patient. The best approach has not yet been defined. Surgical treatment of MUI includes retropubic colposuspension (Burch procedure), a pubovaginal sling, and recently, the new mid-urethral slings (TVT/TOT) which are largely modifications of the pubovaginal sling. Although some recent papers described surgical treatment of MUI, few reports compared different types of anti-incontinence surgery head-to-head. A prospective study randomized 75 women with MUI to anticholinergic agents or surgery (Burch colpususpension vs PVS); 20 patients with pure SUI undergoing surgery served as controls. After 6 months, no significant differences emerged: 87% of women with MUI who underwent Burch were dry compared with 83% after PVS; persistent urge incontinence was similar in the two groups [31].

Several retrospective studies assessed the efficacy of diverse surgical procedures in treating MUI. Gamble et al. recruited 305 women who underwent transobturator, retropubic, or bladder neck sling for MUI and found that transobturator slings had the lowest rate of persistent DO [32]. Rezapour and Ulmsten reported a subjective 85% cure rate in women with MUI after tension-free vaginal tape (TVT) [33]. Although cure rates of MUI after TVT surgery were substantial, they were significantly lower than in women with pure SUI [34]. When comparing first- and second-generation mid-urethral slings, Paick et al. reported on 144 women with MUI who underwent TVT, SPARC, or TOT and found similar cure rates in all three groups for SUI (96%, 90%, and 94%, respectively) and UUI (82%, 86%, and 82%, respectively) [35]. They also reported that preoperative low maximum urethral closure pressure and DO were both associated with increased likelihood of treatment failure of UUI. In a prospective, multi-centre, randomized study comparing TVT and TOT procedures for MUI Kocjancic et al. analyzed 116 consecutive women with stress or mixed UI randomized to TVT (61) or to TOT (55). They showed that with both procedures the most frequent late complication was de novo urgency. Post-operative storage symptoms were not significantly different in the TVT group but were significantly lower in the TOT group. The storage symptom cure rate was 31% after TVT and 55% after TOT [36].

Jain et al. recently reviewed current literature on the impact of mid-urethral slings on MUI symptoms [37••], locating six RCTs and seven prospective non-randomized studies of average to good quality that included women with symptomatic and/or urodynamic MUI. The overall subjective cure rate in these seven studies was 56.4% at 35 months of follow-up. The overall cure of urinary urgency and the UUI component ranged from 30 to 85% with follow-ups ranging from a few months to 5 years; most studies suggested the cure rate waned over time. In a meta-analysis of five RCTs which included women with MUI symptoms, the odds of an overall subjective cure with TVT vs. TOT were similar at 6–33 months follow-up; this finding remained true in a subgroup analysis on women with MUI who did not have urodynamic DO.

The remaining question is whether characteristics of a patient’s urge or UUI can predict surgical failure or success. To provide better information for surgical outcomes attempts were made to define MUI using questionnaire scores that looked separately at Medical, Epidemiological and Social Aspects of Aging (MESA) subscale scores as well as amount of bother according to the Urogenital Distress Inventory (UDI). They were, however, found to be inadequate. Paick et al. reported that the presence of DO was a risk factor for treatment failure for UUI [35]. Schrepferman et al. analyzed a series of 69 patients who underwent PVS for urodynamic SUI with preoperative urge symptoms, 41 with motor urge (DO; further subdivided into 23 patients with low-pressure and 18 with high-pressure urge) and 28 with sensory urge. Urge symptoms were completely resolved in 91.3% of patients with low pressure motor urge compared with 27.8% with high-pressure and 39.3% with sensory urge [38].

Similarly, one may also wonder whether outcomes in women undergoing anti-incontinence surgery with an urge component are worse than outcomes in women with pure SUI. Holmgren et al. evaluated 760 questionnaire respondents who had undergone TVT (112 for MUI, 580 for SUI). Long-term cure rates according to answers to a question regarding incontinence status after surgery as “cured” or “almost cured” were stable at about 85% from 2 to 8 years in the pure SUI group. Cure rates in the MUI group remained around 60% until 4 years, after which they steadily declined to about 30% at 6–8 years [39]. Although the predictive factors of outcome after surgery for MUI remain controversial, multivariable analysis of large series of patients in multi-centre studies could answer our questions in the near future.

## Conclusion

The challenge in identifying an ideal treatment modality for MUI lies in the difficulty in defining it and in lack of consistent coordination between subjective symptoms and urodynamic findings. Furthermore, an effective single treatment may well not even exist. Successful treatment of UUI may make SUI symptoms more prominent and treatment of SUI may be associated with de-novo or persistent UUI symptoms. Although it is clearly not feasible to restrict treatment exclusively to high-volume centres, treatment trends are shifting so that more patients are treated by high-volume providers. This will improve MUI control and decrease women’s dissatisfaction, improving their quality of life.
